# Robotic Sympathectomy for Hyperhidrosis

**DOI:** 10.7759/cureus.33885

**Published:** 2023-01-17

**Authors:** Kapilraj Ravendran, Betsy Babu, Nikolaos Madouros, Nikolaos Panagiotopoulos

**Affiliations:** 1 Medical School, Medical University of Sofia, Sofia, BGR; 2 Thoracic Surgery, St Bartholomew’s Hospital, London, GBR; 3 Thoracic Surgery, University College London Hospitals NHS Foundation Trust, London, GBR

**Keywords:** da vinci, robotic thoracic surgery, hyperhidrosis, sympathectomy, robotic

## Abstract

In hyperhidrosis, the body’s sweat glands overact. Excessive sweating results from this overactivity, and for many hyperhidrosis patients, managing symptoms can be difficult in day-to-day life. Both surgical and non-surgical types of treatment are available for hyperhidrosis. Surgical treatments include microwave sympathectomy (video-assisted thoracic surgery and robotic). Da Vinci Si and Xi robotic systems are used. This review summarizes the outcomes, complications, advantages, and disadvantages of robotic sympathectomy. We conducted a literature search using PubMed, Cochrane, and Scopus. After analyzing nine articles, we found that robotic sympathectomy decreased compensatory hyperhidrosis with similar outcomes to other procedures. Robotic sympathectomy also reduced complications of Horner syndrome and has changed minimally invasive surgery significantly due to the reduction in tremors by a surgeon’s hands to three-dimensional magnified views. It can potentially address the limitations of human video-assisted sympathectomy. However, the higher cost of robotic surgery, longer perioperative time due to the setting up of the machine, and higher training requirements are some of the disadvantages. The advantages of robotic sympathectomy are a reduction in compensatory sweating, better dexterity, loss of tremors, better visualization, and better accuracy. Although the overall success rates seem to be similar between robotic and video-assisted approaches, more studies are needed.

## Introduction and background

Hyperhidrosis is a medical condition characterized by excessive activity of the body’s sweat glands [1]. This results in excessive sweating and malodor [1]. It can be axillary, palmar, plantar, or generalized [2]. The symptomatic management of hyperhidrosis can be a constant challenge [1]. It can affect everyday functioning, causing anxiety and embarrassment in professional and social gatherings [1]. Primary focal (essential) hyperhidrosis is the most common type of this condition [1]. It is characterized by spontaneous overactivity of the nerves responsible for signaling the sweat glands [1]. Despite primary hyperhidrosis being idiopathic, it is most probably linked to a hereditary predisposition [1]. Secondary hyperhidrosis develops on the grounds of an underlying medical condition, such as menopause and hyperthyroidism, or is caused by medication, typically affecting the whole body [1].

There are surgical and non-surgical types of treatment [3]. Non-surgical treatment includes topical antiperspirants (e.g., aluminum chloride), topical lotion, oral medicines (e.g., anticholinergic medicines acting on muscarinic receptors of sweat glands), Iontophoresis, which includes applying a low-intensity electrical current to the hands or feet while immersed in an electrolyte solution, and botox [3]. Surgical treatments include microwave therapy, sweat glands removal, and sympathectomy (video-assisted thoracic surgery (VATS), robotic thoracoscopic approach, thoracoscopic approach, transthoracic approach, transaxillary approach, cervical supraclavicular approach, and posterior thoracic approach) [3].

Ectomy involves the surgical removal of a specified part of the body [4]. Sympathectomy is a medical procedure where the surgeon cuts or clamps the sympathetic nerve [4]. The sympathetic nerve chain travels parallelly up and down along the spine under the deep tissues [4]. It is the part of the nervous system responsible for the fight or flight response [4]. This procedure stops signals from passing down the sympathetic nerve and is used to treat hyperhidrosis [4]. Complications with sympathectomy include bleeding, infection, pain, compensatory sweating, collapsed lung, harming nerves or blood vessels, burning on the underside of arms, and Horner disorder [4]. Positive outcomes of sympathectomy include a much better quality of life and satisfaction rates of 85% after eight months and 74% after 15 years [4]. A complete loss of sweating after two years is expected [4].

In an effort to lessen the frequency and severity of compensatory hyperhidrosis, the sympathetic chain surgical lesion’s extent has been gradually reduced over time [5]. Most people concur that the sympathetic ganglia affected should only contain T3 and T4 [5].

As indicated by the National Institute for Health and Care Excellence guidelines, endoscopic thoracic sympathectomy for craniofacial erythema is an adequate method and functions well enough for use in the NHS; however, the specialists should ensure patients comprehend the risk of serious complications. A very common side effect of the procedure is compensatory hyperhidrosis [6]. Although the pathophysiology of compensatory hyperhidrosis is poorly known, it has been proposed that a malfunctioning reflex arc from the sympathetic nervous system to the hypothalamus may be the primary cause of excessive, sudden, and uncontrolled sweating in other regions of the body [7]. Due to the risk of secondary effects, only patients with extreme perspiring that is affecting their daily existence and who have not reacted to different medicines should consider this procedure [6]. Robotic sympathectomy is a minimally invasive procedure where the sympathetic nerve is burned or clamped using robotics to treat hyperhidrosis [8]. Da Vinci Si and Xi robotic systems are used [8]. Sympathectomies are usually required when a patient presents with symptoms and clinical features such as blushing and flushing as well as hyperhidrosis [9]. Topical medications (aluminum salts), oral anticholinergics (oxybutynin), iontophoresis, or botox injections are insufficiently effective, and the response to therapy is often fleeting [10]. Surgery is typically a more satisfying, almost permanent, and successful form of treatment [10]. It prevents sympathetic impulses from the sympathetic chain from reaching the eccrine sweat glands [10].

In this literature review, we compare the overall success, outcomes, and complications of robotic sympathectomy versus video-assisted sympathectomy. To our knowledge, no recent similar studies have been conducted.

## Review

Methodology

**Figure 1:**
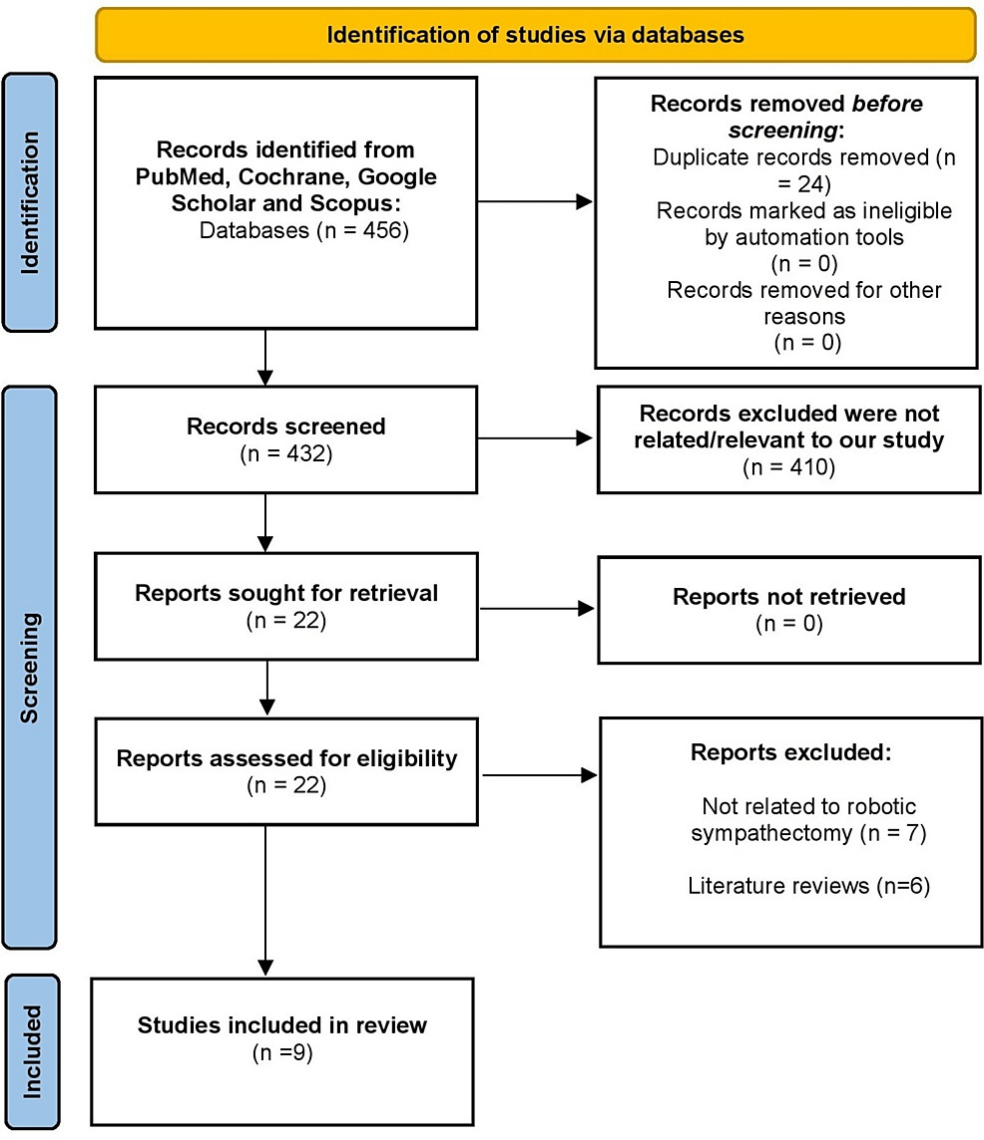
PRISMA flow diagram demonstrating the literature selection strategy. PRISMA = Preferred Reporting Items for Systematic Reviews and Meta-Analyses

We conducted a literature search on PubMed, Cochrane, Google Scholar, and Scopus. Medical Subject Headings (MeSH) terms used were “robotic surgical procedures,” “thoracic surgery,” and “hyperhidrosis.” Abstracts from all articles were obtained and full texts were examined and considered to compare the advantages and disadvantages of robotic sympathectomy for hyperhidrosis. Two authors screened titles and abstracts in English for relevant studies from 2008 to 2022. A total of 24 duplicate articles among the different databases were removed. Our inclusion data was related to robotic surgery used for sympathectomy in the treatment of hyperhidrosis, which were abstracted from each study and included in our review. Articles based on animals and letter editorials were excluded. A total of 22 papers were identified, 13 of which were excluded due to the criteria above. The remaining nine papers were analyzed and summarized, as depicted in the flowchart in Figure 1. Eight papers were case studies/reports, and one paper was a clinical trial. Figure 1 shows our Preferred Reporting Items for Systematic Reviews and Meta-Analyses (PRISMA) flow diagram which explains our literature selection [11].

Results

After excluding secondary sources from the database search and eliminating duplicates, the initial search returned 22 relevant publications. The complete texts of nine publications were evaluated following the screening of titles and abstracts (n = 22) and the exclusion of 13 records due to their lack of relevance to the topic. Finally, nine studies were included in the review. Table 1 lists the papers that were read and examined. The table mainly focuses on outcomes and compensatory hyperhidrosis.

**Table 1:** Studies analyzed in the review with a focus on outcomes and compensatory hyperhidrosis. VATS = video-assisted thoracoscopic surgery; ETS = endoscopic thoracic sympathectomy

Article	Number of patients	Median follow-up	Compensatory hyperhidrosis	Gustatory sweating	Horner syndrome	Operative times	Outcomes and success rates
Coveliers et al. [12]	55 (25 men and 30 women)	24 months	Four (7.2%) patients	One (1.8%) patient	-	Mean 84.3 ± 40.1 (bilateral) minutes	96% had full resolution
Coveliers et al. [13]	25 males and 30 females	-	7% (four out of 55 patients)	-	1 (1.8%) – transient unilateral	Median operative time (bilateral) 80 minutes (ranging from 40 to 260 minutes)	96% had full relief, and 4% had partial relief
Martins Rua, et al. [14]	19 patients were human-assisted group, and 19 patients were voice-controlled robots for holding the endoscopic camera robotic group	Six months	Similar in both VATS and robotic sympathectomy group	-	-	Longer in robotic sympathectomy	Between the two groups, pain, aesthetical results, and the general satisfaction were similar
Connery [15]	Three patients	-	Improved 2/3	-	-	-	Quality of life: 1/3 significantly improved, 1/3 slightly improved, and 1/3 no changes
Chang et al. [16]	Five males, and two females	-	Improved	-	-	The median time of 10.5 hours	No mortality but one patient developed a pneumothorax
Chang et al. [17]	Case report of a 59-year-old female patient	42 months	Significantly reduced	-	-	-	Successful, safe, and precise. Treated the long-term side effects of ETS
Gharagozozloo et al. [8,18]	47 had robotic sympathectomy in a staged fashion (unilateral)	28 ± 6 months	1 (2%) patient	-	1 (2%) patient – transient partial, which resolved in two weeks	67 ± 13 minutes for unilateral robotic selective thoracic sympathectomy	1 (2%) patient had a heart block, and 97% full resolution and relief
Sandhaus et al. [19]	5 males and 8 females with a total of 24 robotic-assisted thoracic sympathectomies	-	Reduced	-	-	106 ± 39 minutes – bilateral robot-assisted thoracic sympathectomy	The cost was 500 euros per case, with no mortality or complications during the hospital stay. The procedure was 100% successful

According to Coveliers et al., robotic thoracoscopic sympathectomy is not only a safe procedure for treating hyperhidrosis but is also effective and feasible with excellent results and low rates of compensatory sweating and complications [13]. With sympathectomy, for palmer hyperhidrosis, axillary hyperhidrosis, and facial flushing, the success rates were 95-97%, 60-80%, and 75%, respectively [13]. In another study by Gharagozloo et al., the intraoperative temperature measurement, patient interviews, and the Hyperhidrosis Disease Severity Scale, which gauges how much sweating interferes with everyday living, were used to assess the success of the sympathectomy [18]. After the sympathectomy, only one patient suffered from compensatory hyperhidrosis [18]. In a study by Sandhaus et al., 13 patients underwent robot-assisted thoracic sympathectomies with a mean age of 30.9 ± 13.0 years (males: five (30.8%), females: eight (69.2%) [19]. There was no in-hospital mortality or conversion to open surgery [19]. Follow-up assessments showed that hyperhidrosis was effectively treated in every case [19]. Results demonstrated that thoracic sympathectomies for individuals with hyperhidrosis can be successfully completed in a safe manner [19].

In a study by Coveliers et al., 55 patients underwent simultaneous bilateral robotic thoracic sympathectomy; entanglements were observed in 1/55 (1.8%) patients, including transient one-sided Horner condition, one-sided dysesthesia of the right hand, transient one-sided secluded ptosis, and bradycardia in 2/55 (3.6%) patients [12]. No chronic Horner disease was noted [12]. Overall, 53/55 (96%) individuals experienced total recovery from hyperhidrosis, while 2/55 (4%) patients only saw partial remission [12]. The mean increase in ipsilateral palmar temperature was 1.2 ± 0.3°C [12] Relief of hyperhidrosis was seen in 98% of patients [12]. In a study by Chang et al. including seven patients with sural nerve grafts, in all cases, the median length was 8 cm and the median operating time was 10.5 hours [16] The median hospital stay was four days [16]. One patient developed palmar hyperidrosis which included the face while another developed pneumothorax [16]. In a case report by the same author, a 59-year-old woman who underwent an endoscopic thoracic sympathectomy (ETS) experienced serious side effects [17]. She displayed emotional anxiety, gustatory hyperhidrosis, overly dry hands, and compensatory hyperhidrosis across the entire trunk [17]. Bilateral sympathetic trunk reversal repair using a Da Vinci Robot and an interpositional sural nerve graft on either side was performed by an interdisciplinary surgical team [17]. The sympathetic trunk stumps and the T2-T4 intercostal nerves were closed with 9-0 sutures to create a microsurgical nerve graft [17]. Palmar dryness and emotional anxiety were significantly reduced at 24, 33, and 42 months [17].

Discussion

After assessing the literature, we analyzed the advantages and disadvantages of using the robotic approach for sympathectomy for hyperhidrosis, and Table 2 summarizes our findings.

**Table 2:** The advantages and disadvantages of robotic sympathectomy for hyperhidrosis.

Advantages	Disadvantages
Decrease in rates of conversion compared to open operations [15]	Cost [12,13,19]
Decrease in compensatory hyperhidrosis [15]	Longer operative times and longer setup times [20]
Decrease in bleeding and clearer incisions along with similar outcomes and shorter learning curves [15]	Fewer people are familiar with robotic systems, which require training [12,13]
Three-dimensional visualization and seven degrees of freedom [12,13,15,20]	No touch sensation [20]
Improved dexterity and elimination of physiologic tremors and fulcrum effect [12,13,15]	Extra staff to operate the systems [12,13]
Less blood loss and decrease in complications as well as nerve preservation [12,13,15]	

ETS usually leaves a patient with complications such as compensatory sweating noted in more than half of patients, while robotic sympathectomy reduces these complications [9,15,21]. A patient’s biggest doubts regarding surgery are the side effects, in particular, excessive compensatory hyperhidrosis and anhidrosis [21,22]. Therefore, patients in the past have opted for pharmacological treatments [22]. Treating compensatory sweating after sympathetic nerve clipping is impossible as the procedure is irreversible, such that patients have to use botulinum injections [23]. In some cases, the use of botulinum can be more beneficial than ETS [24]. Treatments are needed as hyperhidrosis has been shown to have psychosocial effects on patients [25]. Studies have shown that sympathectomy can help significantly with these effects [26]. Surgical interventions for sympathectomy have been proven to be safe as well as efficient and effective [27]. Sural nerve grafting has reported improvements in patient outcomes [28]. Studies also suggest robotic surgeries are the future of sympathectomy [29].

A study by Martins Rua et al. showed that there was no major difference regarding the majority of safety criteria in both the human-assisted and robotic-assisted groups; however, incorrect camera movements were less common in the robotic-assisted group [14]. Similar studies examined the use of robotics in video-assisted thoracic sympathectomy (VATS) by comparing a robotic versus a human-held camera [12,13]. The study showed that the robotic approach used four incisions instead of two, the patient needed to be rotated 180 degrees under general anesthesia, longer operative time, and higher cost but there was an improved three-dimensional view [12,13]. The costs of robot-specific one-time material were estimated in another study to be 500 euros per case [12,13,19].

The use of a robotic arm lengthens surgery, according to a study by Kondraske et al. The study showed that while other studies find that it shortens surgery by six times, it is dependent on the surgeon’s learning curve [20]. The shifting of robotic instruments from left to right during procedures was presumably another element that prolonged the duration [20]. Despite the fact that using robotic equipment for routine surgical procedures does not offer significant advantages, doing so improves the learning curve by giving the surgical team more experience and expertise [20]. We feel that this is the unavoidable future of some thoracic surgery operations as these advancements can be used in more intricate surgical procedures using more complex robotic instruments [20]. Overall, although having faster learning curves and being as safe as human-holding during a VATS sympathectomy, robotic camera handling lengthened the surgical procedure [20].

Nerve graft reconstruction should be made available to patients with severe complications or side effects from sympathectomy for hyperhidrosis who have used up all available options for treatment as a chance for them to be able to reverse these negative symptoms and experience measurable improvements in their quality of life [15]. Sympathetic nerve regeneration has been proven in experimental models and clinically, according to a group study by Connery [15]. A platform for carrying out this nerve reconstruction with a minimally invasive method is provided by the Da Vinci robotic nerve graft reconstruction [15]. It enables typical nerve graft regeneration and offers excellent imaging, dissection, and neurolysis using microsurgical instruments at high magnification [15]. The outcomes showed that the Da Vinci robot’s tremor filtration and high-magnification camera made it possible to manipulate a 2 mm wide nerve graft and 10-0 monofilament suture [15]. This demonstrates its efficacy in enhancing magnification and lowering tremor [15].

Another study noted that the three-dimensional, self-controlled 10-fold magnified view offered by the robotic camera allowed for better visualization, enabling easier identification of the healthy proximal stump of the sympathetic trunk [16]. Second, even in a small anatomical region, robot-assisted surgery makes it possible to meticulously suture with 8-0 nylon [16]. Intercostal nerves were not used in the procedure; instead, a sural nerve graft was used. Even with a small visual field, it is difficult to harvest the intercostal nerves for an adequate amount of time [16]. Another study with comparable findings highlighted the usage of articulated micro-instrumentation designed to have seven degrees of freedom to accomplish this [17]. A precise, secure, and effective treatment for ETS sequelae may be offered in a highly specialized interdisciplinary environment [17]. This indicates the therapeutic viability of treating compensatory hyperhidrosis or other unfavorable outcomes from sympathectomy as long-term side effects following ETS [17]. Additionally, the sural nerve’s larger caliber permits more end-to-side coaptation to the intercostal nerves, preserving the original structure [17]. Results are improved and should be highlighted by a microsurgeon with experience in coaptation and peripheral nerve dissection [17]. Fundamental pillars for quality, safety, and dependability include the sensitive identification of a healthy nerve stump and a dependable procedure [16,17].

Limitations

The majority of the studies were case reports. As a few papers had the same author, future studies with different authors will provide more outcomes with a larger study population. The same authors wrote four papers used in our review [8,12,13,18]. One author also wrote two papers in our review [16,17]. Control case studies should also be conducted to prove and enhance our results further. More clinical trials and systematic reviews are needed to assess the long-term results. As some studies were also limited to a few patients, more trials including more patients are needed to assess the benefits of robotic sympathectomy.

## Conclusions

The overall success rates are the same as sympathectomy. Robotic sympathectomy is promising as it has similar outcomes and decreases the incidence of compensatory sweating. Studies have shown that perioperative times are longer due to more time needed to set up the robot. However, precision, magnification, dexterity, and viewing are significantly enhanced via robotics, which are major advantages along with shorter learning curves. However, one hindrance proved to be the cost of the procedure, which needs to be examined to make it more accessible to multiple centers. This study provides the groundwork for future studies as our analysis showed reduced complications in treating hyperhidrosis when using the robotic approach.
